# The prognostic significance of ubiquitination-related genes in multiple myeloma by bioinformatics analysis

**DOI:** 10.1186/s12920-024-01937-0

**Published:** 2024-06-19

**Authors:** Feng zhang, Xiao-Lei Chen, Hong-Fang Wang, Tao Guo, Jin Yao, Zong-Sheng Jiang, Qiang Pei

**Affiliations:** 1 Department of Hematology, Kunming First People’s Hospital, Kunming, 650051 China; 2 Department of Endocrinology, Kunming First People’s Hospital, Kunming, 650051 China; 3 Multidisciplinary Diagnosis and Treatment Center for Oncology, The Second Affiliated Hospital of Kunming Medical University, Kunming, 650101 China; 4 Department of Hematology, The First People’s Hospital of Yunnan Province, Kunming, 650032 China

**Keywords:** Multiple myeloma, Ubiquitination-related gene, Ubiquitin-proteasome system, Risk model, Prognosis

## Abstract

**Background:**

Immunoregulatory drugs regulate the ubiquitin-proteasome system, which is the main treatment for multiple myeloma (MM) at present. In this study, bioinformatics analysis was used to construct the risk model and evaluate the prognostic value of ubiquitination-related genes in MM.

**Methods and results:**

The data on ubiquitination-related genes and MM samples were downloaded from The Cancer Genome Atlas (TCGA) and Gene Expression Omnibus (GEO) databases. The consistent cluster analysis and ESTIMATE algorithm were used to create distinct clusters. The MM prognostic risk model was constructed through single-factor and multiple-factor analysis. The ROC curve was plotted to compare the survival difference between high- and low-risk groups. The nomogram was used to validate the predictive capability of the risk model. A total of 87 ubiquitination-related genes were obtained, with 47 genes showing high expression in the MM group. According to the consistent cluster analysis, 4 clusters were determined. The immune infiltration, survival, and prognosis differed significantly among the 4 clusters. The tumor purity was higher in clusters 1 and 3 than in clusters 2 and 4, while the immune score and stromal score were lower in clusters 1 and 3. The proportion of B cells memory, plasma cells, and T cells CD4 naïve was the lowest in cluster 4. The model genes KLHL24, HERC6, USP3, TNIP1, and CISH were highly expressed in the high-risk group. AICAr and BMS.754,807 exhibited higher drug sensitivity in the low-risk group, whereas Bleomycin showed higher drug sensitivity in the high-risk group. The nomogram of the risk model demonstrated good efficacy in predicting the survival of MM patients using TCGA and GEO datasets.

**Conclusions:**

The risk model constructed by ubiquitination-related genes can be effectively used to predict the prognosis of MM patients. KLHL24, HERC6, USP3, TNIP1, and CISH genes in MM warrant further investigation as therapeutic targets and to combat drug resistance.

**Supplementary Information:**

The online version contains supplementary material available at 10.1186/s12920-024-01937-0.

## Introduction

Multiple myeloma (MM) is a prevalent hematological malignancy characterized by the malignant proliferation of plasma cells in the bone marrow. This results in the secretion of monoclonal immunoglobulin or its fragments, leading to bone marrow failure, multiple bone destruction, and damage to various organs or tissues [1]. The number of MM patients is increasing worldwide [2]. The incidence is about 1.03% in China, and there are more male patients than female patients [3]. However, the etiology of MM is still unclear. Most researchers agree that abnormal plasmacytes originate from memory B lymphocytes or proplasmacytes with C-myc gene recombination and high expression levels of certain N-ras genes.This leads to unrestricted plasmacyte proliferation and abnormal increase of IL-6 in the bone marrow. The clinical manifestations include anemia, kidney impairment, hypercalcemia, and other symptoms, while severe cases may lead to death [4]. Therefore, exploring the genes associated with MM will help provide a theoretical basis for early diagnosis and treatment targets.

The ubiquitin-proteasome system (UPS) plays a critical role in the therapy of MM [5]. The inhibition of UPS has been considered a new strategy against MM. For example, the proteasome inhibitor (PI) can reduce the expression of TNF-α and NF-κB, induce apoptosis, and suppress drug efflux [6]. Immunomodulatory drugs are mainly applied to the treatment of MM, targeting the UPS. Immunomodulatory drugs induce selective ubiquitination and degradation of MM-associated lymphatic transcription factors IKZF1 and IKZF3 by binding to E3 ubiquitin ligase substrate receptor protein targets (CRBN) [7, 8]. Neuronally expressed developmentally downregulated 4 − 1 (NEDD4-1) inhibits the AKT signaling pathway by inducing proteasome degradation through ubiquitinating phosphorylated AKT-Ser473 and promoting apoptosis of MM cells. The PI Bortezomib can lead to the accumulation of polyubiquitylated proteins. Bortezomib promotes apoptosis of MM cells by inhibiting the 26 S proteasome [9]. Anti-MM drugs show their activity mainly by inhibiting ubiquitin-related enzymes, and target ubiquitin pathway promotes MM cell death. But drug resistance often develops [10]. The evidence showed that hyperactive small ubiquitin-like modifier is closely related to the progression of MM, while the higher the level of hyperactive small ubiquitin-like modifier, the poorer the survival [11, 12]. The research by Du et al. indicated that the ubiquitin receptor PSMD4/Rpn10 is effective in regulating cytotoxicity in MM, making it a potential therapeutic target [13]. According to the findings, it can be seen that ubiquitination is closely related to the development and treatment of MM. Although immunomodulatory drugs can prolong survival, there is still the possibility of relapse and drug resistance [14]. Moreover, PI showed serious side effects, causing cardiovascular damage [15]. Hence, there is an urgent need to develop new targets to treat MM and reduce drug resistance.

## Methods

### Download expression data and ubiquitination-related genes

We employed the TCGA database to download gene expression data and clinical information of MM as a training dataset of the risk model under the project ID MMRF-COMMPASS by using the R statistical software (version 4.2.1, https://mirrors.tuna.tsinghua.edu.cn/CRAN/bin/windows/base/R-4.2.1-win.exe) based on R package GDCRNATools (v1.16.2, https://github.com/Jialab-UCR/GDCRNATools, access time: 2023-6-20) [16]. A total of 859 samples were included in the TCGA MMRF-COMMPASS dataset. Among them, 764 MM samples were selected as a case group by PrimaryBloodDerivedCancer-BoneMarrow screening. Then, we used BoneMarrowNormal with project ID TARGET-AML downloaded from the leukemia dataset to screen normal bone marrow tissue samples due to the absence of normal samples in the MMRF-COMMPASS dataset. As a result, we got 20 normal samples for the control group. The expression data from 784 integrated samples were used for subsequent analysis. In the meanwhile, we obtained a gene expression dataset of MM samples with prognostic information GSE2658 [17] (549 tissue samples of MM at https://www.ncbi.nlm.nih.gov/geo/query/acc.cgi?acc=GSE2658, Chip platform GPLGPL570), probe data and survival information necessary to verify the risk model. We searched for the keyword “ubiquitin” to acquire the gene set from the Signatures Database [18] v7.5.1 (http://www.gsea-msigdb.org/gsea/msigdb/index.jsp). A total of 197 C2 curated sets were obtained, and 1282 related genes were selected after merging data and de-duplicating for subsequent analysis.

### Analysis of MM differentially expressed genes (DEGs) and differentially curated ubiquitination-related genes and identification of ubiquitination-related subtypes

The analysis of DEGs was performed based on MM expression data and normal bone marrow expression data by using the limma method [19] of the built-in gdcDEAnalysis function in the GDCRNATools package. In addition, we conducted an intersection between DEGs and curated ubiquitination-related genes. In order to conduct a more accurate model, individual differences in ubiquitination gene expression were excluded. We applied R package ConsensusClusterPlus (http://bioconductor.org/packages/release/bioc/html/ConsensusClusterPlus.html) based on the expression quantity of differentially curated ubiquitination genes to conduct consistent cluster analysis on the samples of the training dataset and set parameters: maxK = 10 (maximum number to evaluate), reps = 100 (number of subsamples), while the most appropriate cluster number was selected, the samples were divided into different subtypes, and the classification reliability was verified by PCA.

### K-M survival analysis among different ubiquitination subtypes, analyzing tumor immune infiltration analysis, and comparison of clinical information differences

The survival differences between different ubiquitination subtypes were analyzed. The Estimate algorithm [20] was employed to perform immune scores for samples of each subtype and to compare the differences in immune scores. Moreover, CIBERSORT [21] was used to evaluate the proportion of immune cell infiltration and compare the difference in samples of each subtype.

### Differences in checkpoint genes and HLA family genes between subtypes and representative gene collections

The list of immune checkpoint genes was obtained based on published article [22], and the list of HLA family genes was obtained by searching the HLA nomenclature database (http://hla.alleles.org/genes/index.html). Wilcoxon test was used to compare the expression of immune checkpoint genes and HLA family genes among subtypes.

For obtaining representative genes of subtypes, we used the limma method from the R package to conduct difference analysis between samples of a specific subtype and samples of other subtypes. Only genes highly expressed in the subtype (logFC > 1, *P* < 0.05) were selected as representative genes of the subtype.

Prognostic risk models were constructed based on single-factor and multiple-factor analysis [23] and scored after picking the union set de-duplicating representative genes for each subtype and collecting the survival information of each subtype.

### The predictive power evaluation of the risk model based on the TCGA dataset

According to the median risk score, the samples were divided into a high-risk group and a low-risk group, and the survival difference between the high- and low-risk groups was compared respectively. Finally, the ROC curve was plotted.

The independent predictive power of the risk model was evaluated based on a nomogram and a calibration graph combined with the risk model score and clinical phenotype.

### Risk model validation based on the GSE2658 dataset

According to the risk model, risk scores were assigned to the samples in the validation dataset, and the survival difference between high-risk and low-risk samples was compared using the median as the standard. The predictive power of the validation dataset is verified by drawing the nomogram and a calibration graph simultaneously.

### Prediction of drug sensitivity and subtype characterization correlation analysis

We utilized the Genomics of Drug Sensitivity in Cancer (GDSC; https://www.cancerrxgene.org/) database to estimate the sensitivity of each patient to chemotherapy drugs. The half maximal inhibitory concentration (IC50, where a lower IC50 value indicates greater cell sensitivity to the drug) was quantified using the pRRophetic package in the R language. The Wilcoxon test was used to compare drug sensitivity between high- and low-risk groups. The classification samples of different subtypes within the high- and low-risk groups were collected and analyzed.

### External validation of model genes

The Kaplan-Meier Plotter was used to verify the correlation between model genes and MM survival. The *P*-value < 0.05 was considered statistically significant.

## Results

### DEGs analysis

**Fig. 1:**
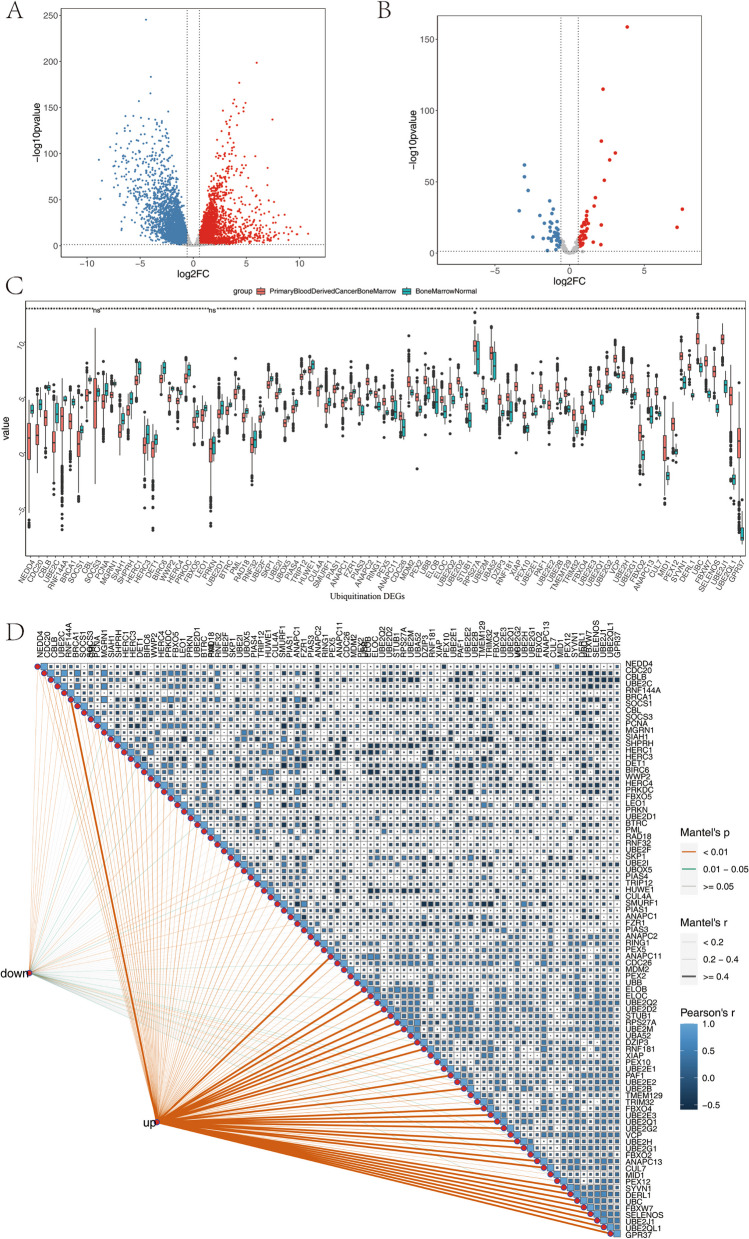
** A** DEGs volcano map of MM and normal bone marrow samples; **B** Ubiquitination-related DEGs volcano of MM and normal bone marrow samples; **C** There were significant differences in ubiquitination-related genes; **D** Correlation analysis of ubiquitination-related DEGs. *: 0.01 < *P* < 0.05, **: 0.001 < *P* < 0.01, ***: 0.0001 < *P* < 0.001, ****: *P* < 0.0001

A total of 784 samples were obtained after screening for subsequent analysis. In the meanwhile, we obtained 197 ubiquitination-related genes from the Signatures Database. A total of 6533 DEGs were identified using the limma method with |log2 (FC)| > 0.58 and *P* < 0.05 as the criterion through the built-in gdcDEAnalysis function of the GDCRNATools package (Fig. 1A). Furthermore, we got 87 differential ubiquitination-related genes. Among them, 47 genes were highly expressed in the tumor, and 40 genes were highly expressed in the control group, suggesting a close relationship between ubiquitination-related genes and MM (Supplementary Table 1, Fig. 1B). The expression levels of ubiquitination-related genes are presented in Supplementary Table 2. The results of the Wilcoxon test were statistically significant (Fig. 1C). The correlation analysis of ubiquitination-related DEGs was shown in Fig. 1D. The results suggest a strong correlation among highly expressed ubiquitination-related genes.

### Identification of molecular subtypes

**Fig. 2:**
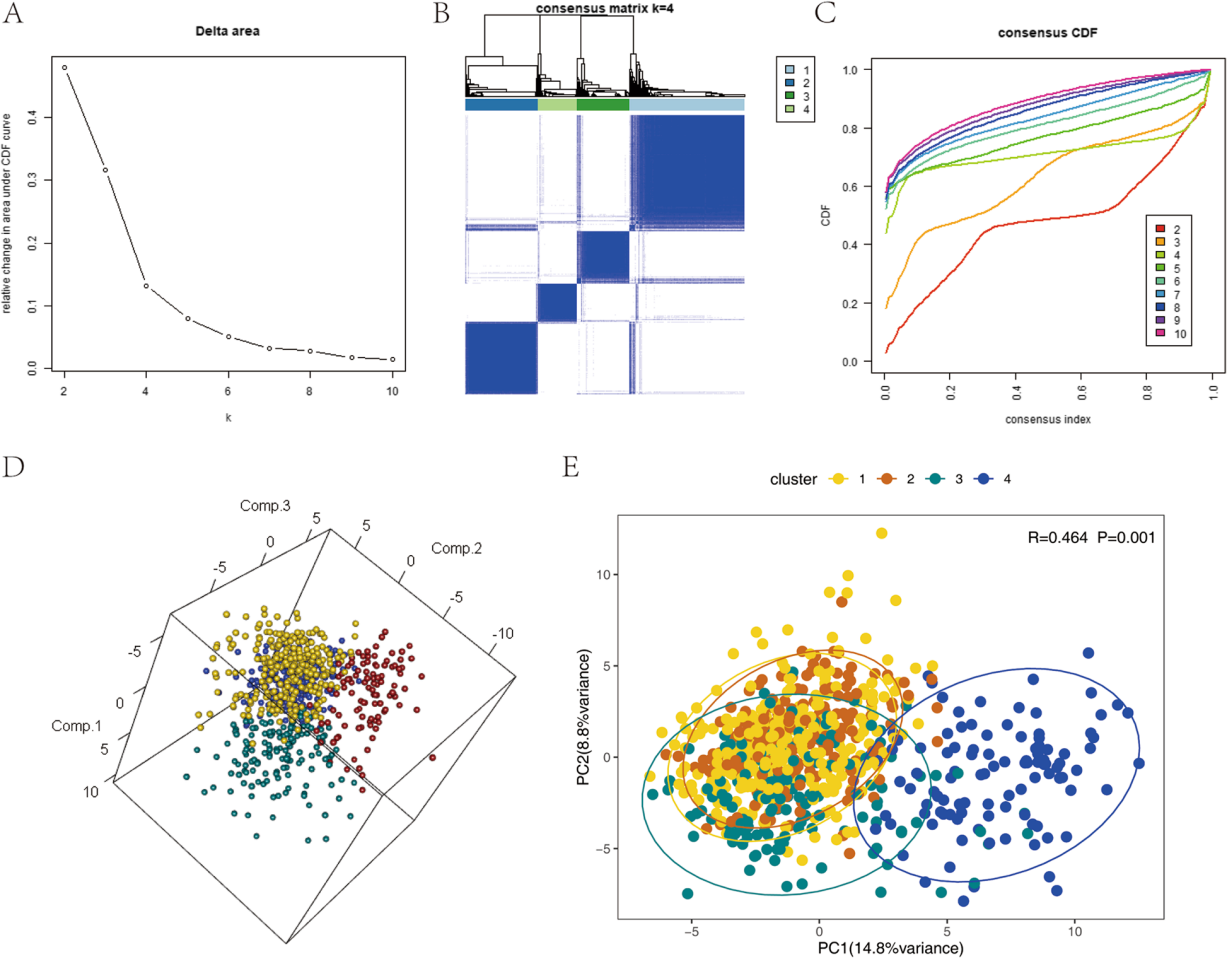
** A** Change of area of CDF curve under the number of clusters; **B** Clustering hierarchy diagram; **C **CDF curve for each cluster number; **D** PCA of differentially ubiquitination-related genes in 3D; E PCA Anosim in 2D, *R* > 0 refers there is difference between clusters, *P *< 0.05 refers the difference is statistically significant

According to the method, we performed a consistent cluster analysis of 764 TCGA MM samples based on the 84 ubiquitination-related genes obtained earlier. We selected the Delta area to show the inflection point (Fig.2A), and the clustering heat-map was well-structured (Fig. 2B), while the CDF curve was leveling off (Fig.2C). The corresponding k value was used as the number of clusters, so we finally determined that the number of clusters is 4, that is, the samples are divided into four subtypes. Principal Component Analysis (PCA) was conducted using the expression levels of ubiquitination genes, followed by ANOSIM analysis to compare thevariations among subtypes (Fig. 2D-E).

### K-M survival analysis between different immune subtypes, analyze immune cell infiltration, and correlate with clinical data

**Fig. 3:**
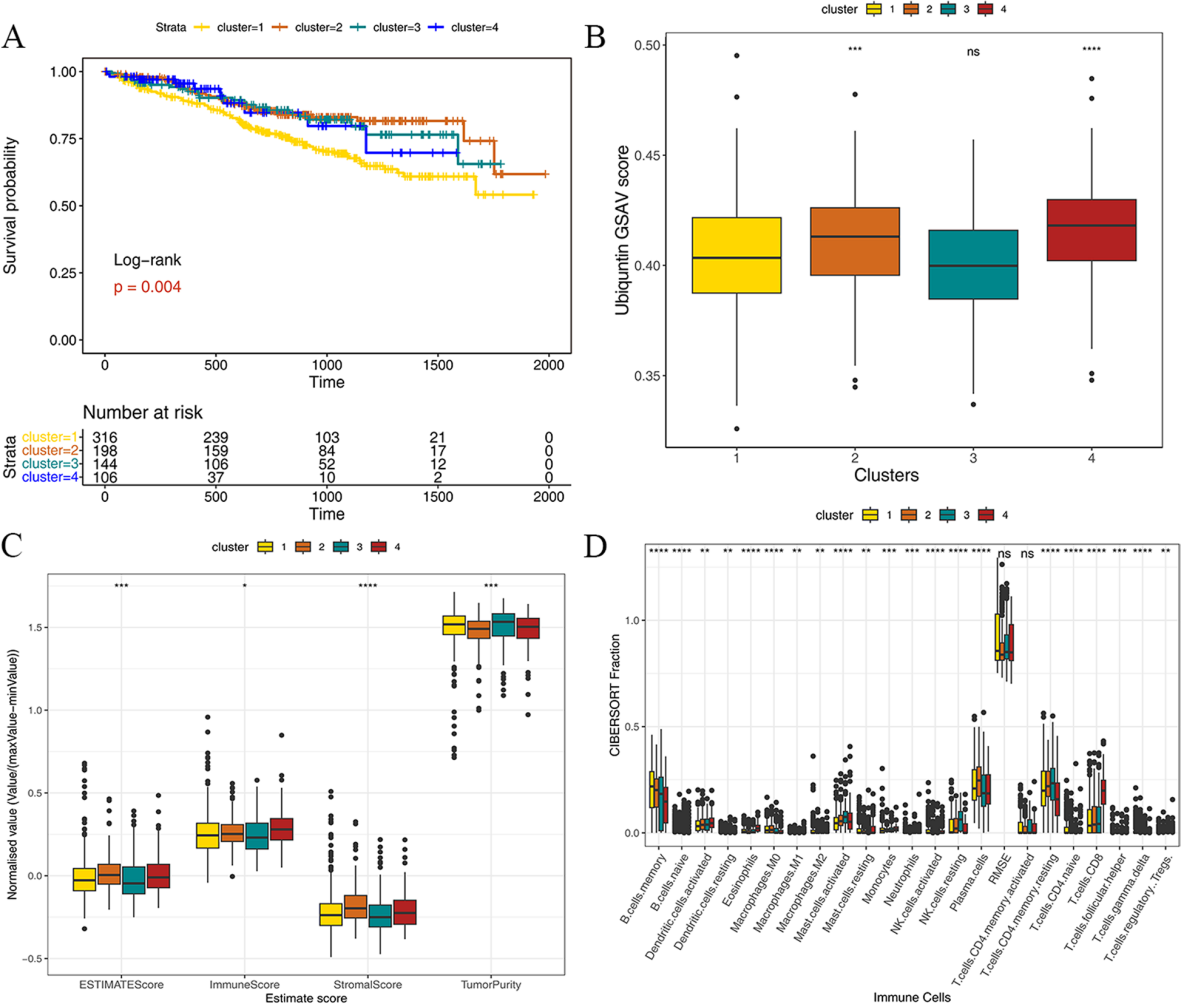
** A** The differences in survival analysis in ubiquitination-related gene clusters; **B **The differences in enrichment scores in ubiquitination-related gene clusters; **C **The differences of immune infiltration scores in ubiquitination-related gene clusters; **D **The differences in the proportion of immune cells in ubiquitination-related gene clusters. Wilcoxon Test *: 0.01 < *P *< 0.05, **: 0.001 < *P *< 0.01, ***: 0.0001 < *P *< 0.001, ****: *P *< 0.0001

Based on the survival information of samples in each subtype, we utilized the R packages survival and survminer to compare the survival conditions of different subtypes.Our analysis revealed significant differences in survival between subtypes (Fig. 3A). Among them, cluster 1 had a poor prognosis, while the rest had a better prognosis. At the same time, the results of enrichment scores showed that the score of cluster 1 was significantly lower than that of cluster 2 and cluster 4 (Fig. 3B), which illustrated that ubiquitination-related genes were related to the prognosis of MM patients. In addition, the immune scores were performed by using the ESTIMATE algorithm, and we compared the differences in immune scores among subtypes (Fig. 3C). As a result, the differences in immune scores between the subtypes were statistically significant. The tumor purity of cluster 1 and cluster 3 was significantly higher than those the other two clusters. The immune score and stromal score in cluster 2 and cluster 4 were higher than in cluster 1 and cluster 3. The GSVA score of each cluster was consistent with the ESTIMATE score, which confirmed the correlation between ubiquitination-related genes and MM pathogenic progression. The proportion of immune cells in tumor tissues, evaluated by the CIBERSORT algorithm showed that the differences in CIBERSORT fraction in clusters were statistically significant (Fig. 3D). In terms of immune cells, B cells memory in cluster 1 accounted for the highest proportion, while the proportion of plasma cells in cluster 1 was less than in cluster 2. Additionally, T cells CD8 accounted for the highest proportion in cluster 4.

### The collection of representative genes in each cluster

**Fig. 4:**
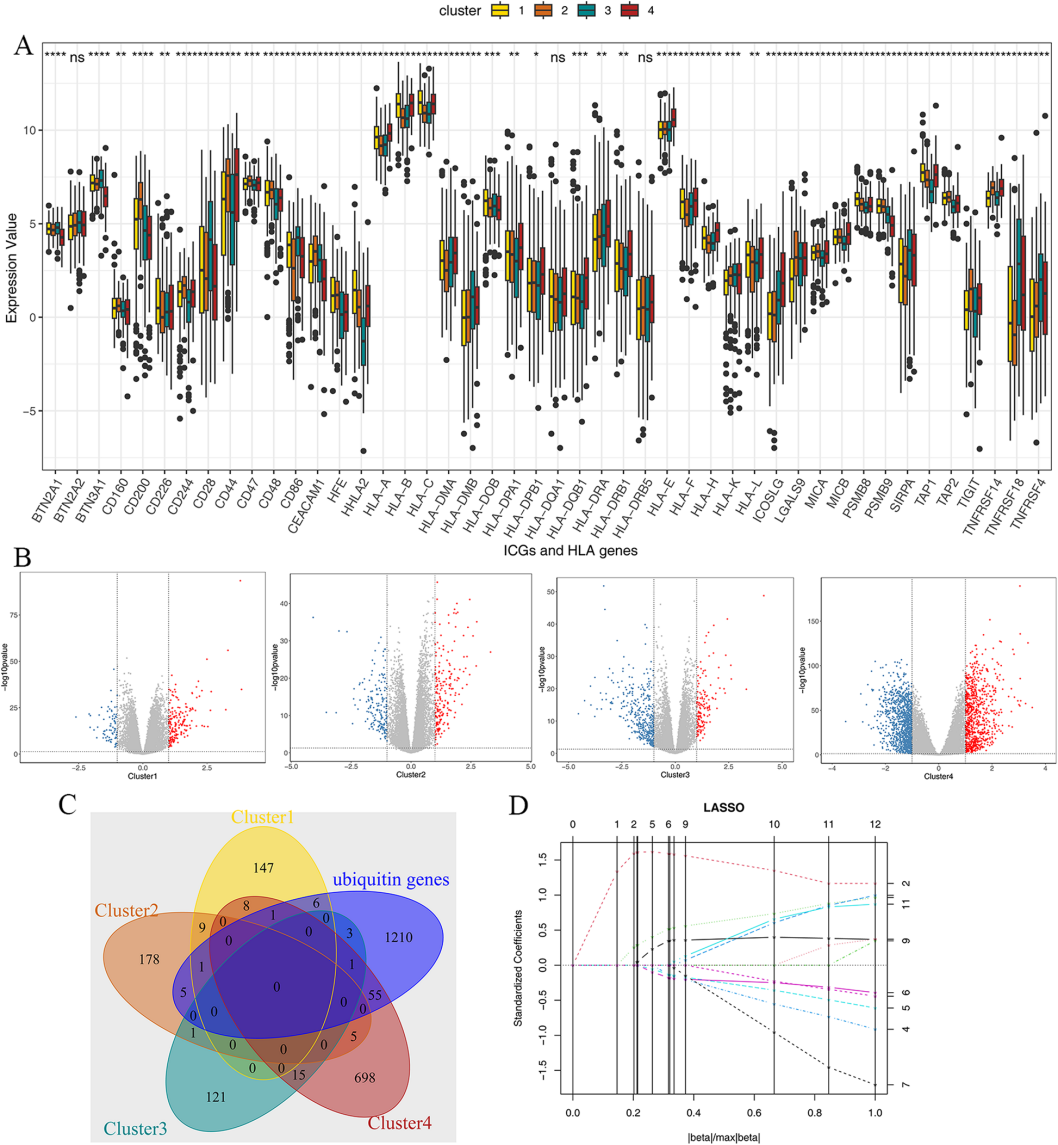
** A** The differences between checkpoint genes and HLA family genes in subtypes. (Wilcoxon Test *: 0.01 < *P* < 0.05, **: 0.001 < *P* < 0.01, ***: 0.0001 < *P* < 0.001, ****: *P* < 0.0001); **B** The representative genes of clusters (The red dots represent the representative genes of each cluster, log2FC > 1 and *P* < 0.05); **C **The veen diagram of representative genes in each cluster; **D **LASSO regression

The expression levels of checkpoint genes and HLA family genes are presented in Supplementary Table 3. The results revealed significant differences in checkpoint genes and HLA family genes among various molecular subtypes, indicating notable distinctions significant differences between the subtypes. For example, the expression level of BTN3A1 in cluster 4 was lower than in other clusters, and the expression level of HLA-A, B, and C in clusters 1 and 4 was higher than in clusters 2 and 3 (Fig. 4A). Moreover, we utilized the limma package to analyze the differences between the samples of one subtype and other subtypes, and selected the highly expressed genes as representative genes of each subtype (Fig. 4B). The intersection of representative genes in subtypes was limited, meaning that the differences in representative genes of each subtype were significant. In the meanwhile, we selected all sets of ubiquitination-related genes in the Signatures Database to intersect with all subtypes, and we got 72 ubiquitination representative genes for subsequent survival analysis (Fig. 4C). Then, we constructed a risk model combined with the survival information of the samples. The reason LASSO regression only considers survival and death time, and does not include survival information, is that we need to select genes that are significantly related to survival time and survival state in the single-factor analysis to ensure the reliability of the model. Based on single-factor analysis, the LASSO regression was performed on genes with *P* < 0.005 (Fig. 4D).

### The risk model construction and validation based on the TCGA dataset

The risk score model was constructed according to the results of LASSO model: Riskscore = 0.0046833357739203*SOCS3 + 0.0296897767259966*UBE2T + 0.0185158927170784*TOP2A + (-0.0189779895672147)*HERC6 + (-0.0261853093234728)*USP3 + (-0.0124611715684952)*TNIP1 + (-0.0668058378550144)*KLHL24 + 0.0114569251299551*SDE2 + 0.0439772194879231*VCPIP1 + 0.0270466928975837*YOD1 + (-0.00796472303046347)*CISH”.

**Fig. 5:**
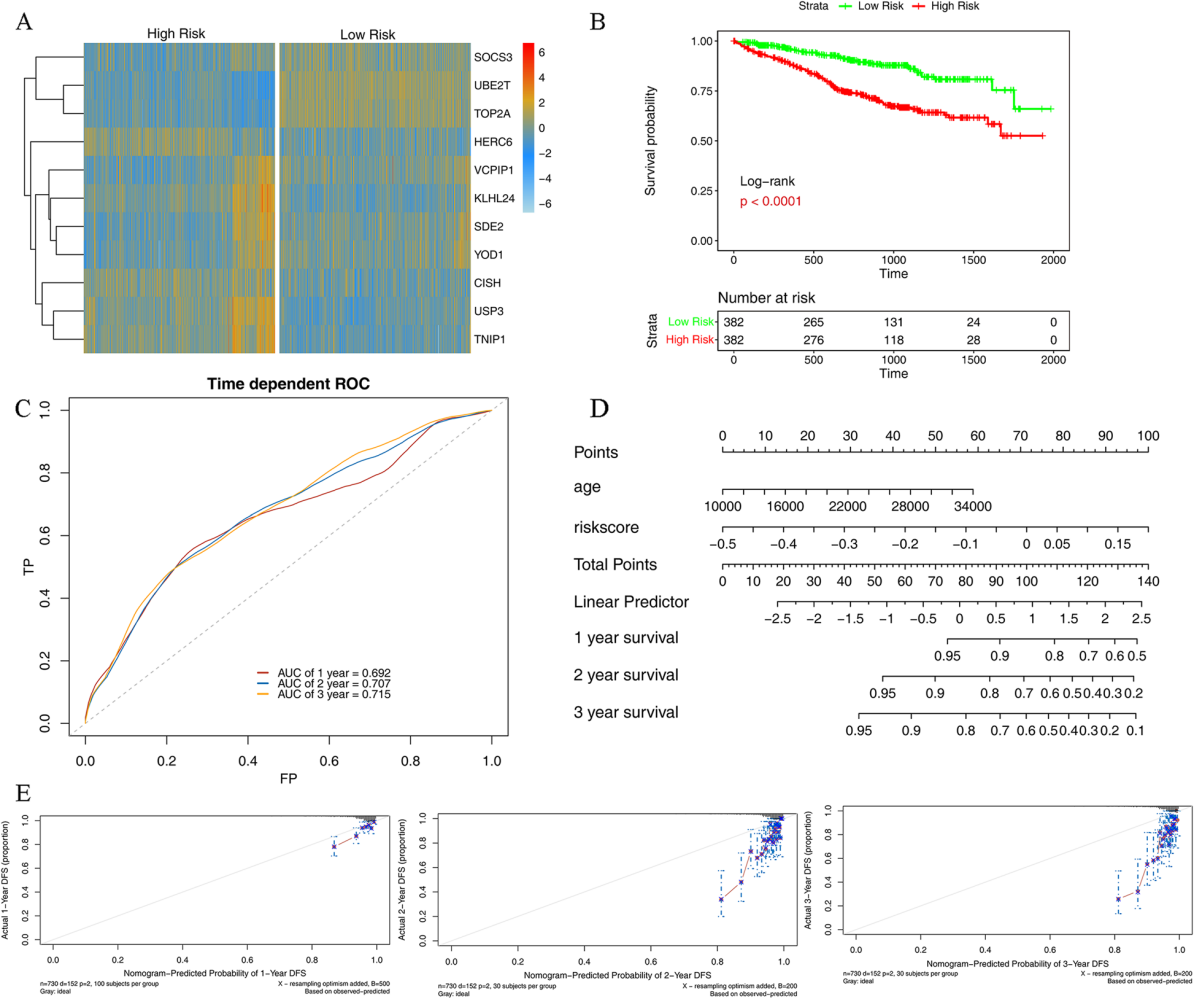
** A** The heat map of gene expression in low- and high-risk groups; **B **The survival probability of low- and high-risk groups; **C** The ROC curve of the risk model in the TCGA dataset; **D** Nomogram of risk model; **E** The calibration graph of 1-, 2- and 3-year survival rate

After scoring the TCGA MM samples, the samples were divided into a high-risk group and a low-risk group according to the median risk score. The differences in gene expression (Fig. 5A) and survival probability (Fig. 5B) between high- and low-risk groups were statistically significant, while the ROC curve had a high interpretive ability (Fig. 5C). The model genes UBE2T, TOP2A, VCPIP1, SDE2, YOD1, and SOCS3 were highly expressed in the low-risk group, while KLHL24, HERC6, USP3, TNIP1, and CISH were highly expressed in the high-risk group. Based on the prognostic model and TCGA dataset, we conducted univariate and multivariate analyses with independent prognostic clinical information and risk score, and found that age and risk score were significantly correlated with prognostic survival. According to the age and risk score, the risk model nomogram (Fig. 5D) and calibration graphs of 1-, 2-, and 3-year survival rates were conducted (Fig. 5E).

### The validation of the risk model based on the GSE2658 dataset

**Fig. 6:**
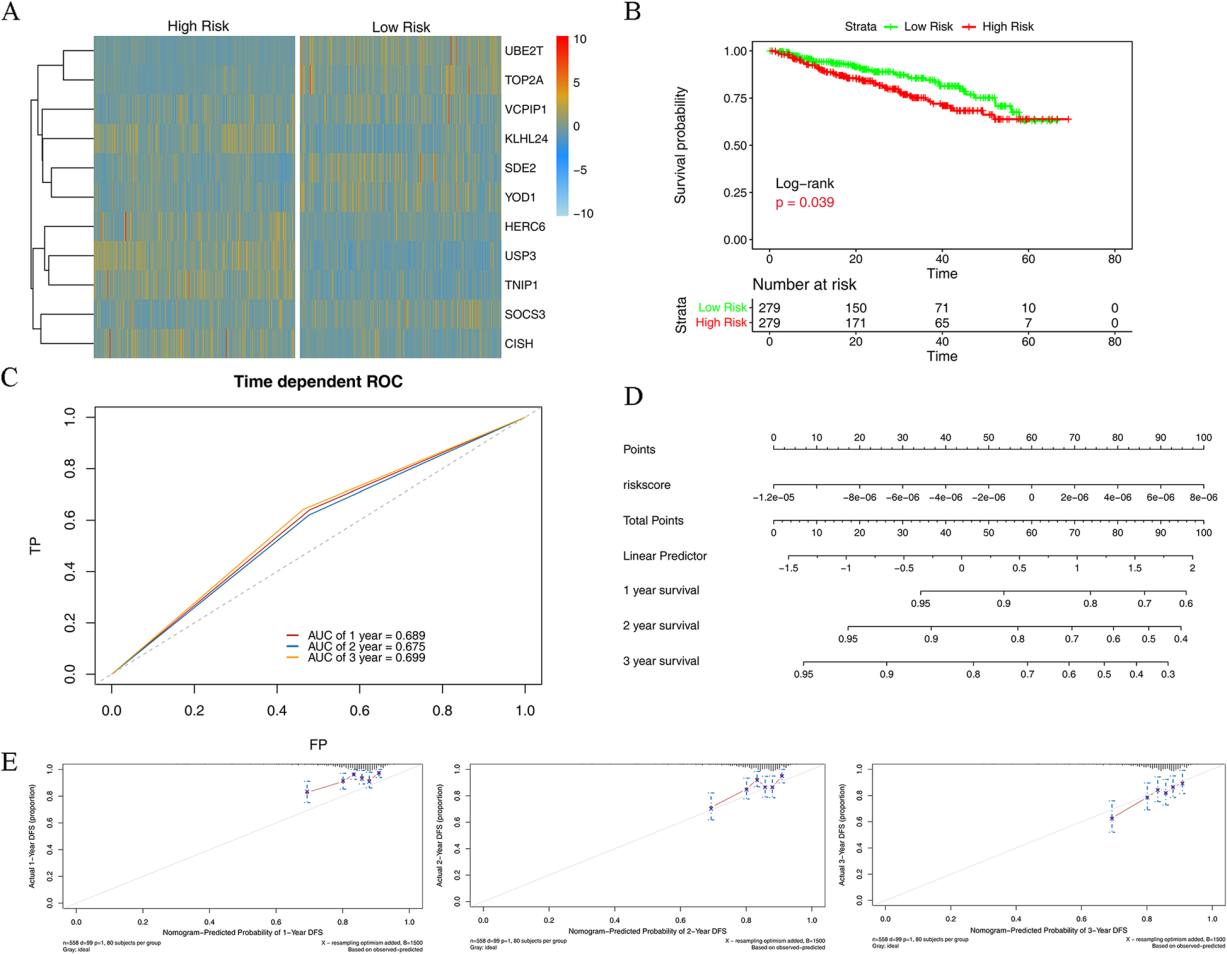
** A **The expression of model genes in high- and low-risk groups based on the GEO validation dataset; **B **The survival analysis of model genes in high- and low-risk groups based on the GEO validation dataset; **C **The ROC of risk model prediction in high- and low-risk groups based on validation dataset; **D **The nomogram of validation dataset; **E **The calibration graphs of 1-, 2- and 3-year survival model based on GEO dataset

Based on the expression of model genes in the validation dataset GSE2658, we scored the risk of MM samples (Supplementary Table 4), and divided them into high- and low-risk groups according to the median risk score. According to the expression of model genes in the high- and low-risk groups (Fig. 6A), the model genes UBE2T, TOP2A, VCPIP1, SDE2, YOD1, and SOCS3 were highly expressed in the low-risk group, while KLHL24, HERC6, USP3, TNIP1, and CISH were highly expressed in the high-risk group. In addition, survival analysis was conducted for the samples in high- and low-risk groups (Fig. 6B). The survival probability was higher in the low-risk group than in the high-risk group, and the difference was statistically significant. The ROC curve indicated that the risk model effectively predicted the prognosis of MM (Fig. 6C).

The results of single-factor analysis indicated that the risk score was significantly correlated with survival time (Supplementary Table 5). The higher the risk score, the shorter the survival time. The nomogram (Fig. 6D) and calibration graphs of 1-, 2- and 3-year survival rates (Fig. 6E) were plotted.

### The prediction of drug sensitivity and subtype characterization correlation analysis

**Fig. 7:**
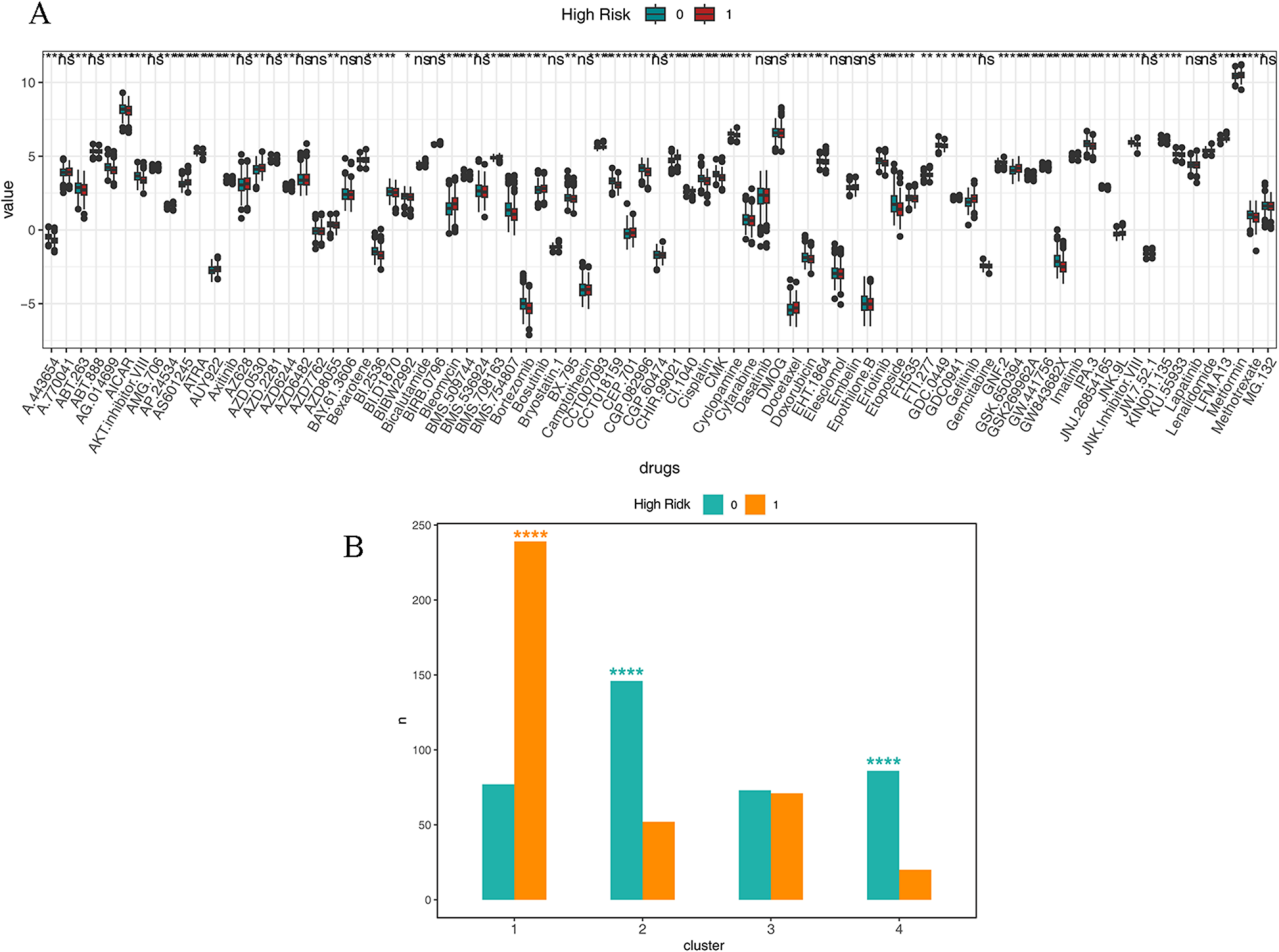
** A** The prediction of drug sensitivity in the TCGA dataset and the difference in high- and low-risk groups; **B **The enrichment analysis of high- and low-risk groups in the TCGA dataset

Based on the TCGA MM sample expression dataset and GDSC database to estimate the sensitivity to chemotherapy drugs for each patient, and compare the drug sensitivity between high- and low-risk groups. Most drugs had significantly different sensitivity in high- and low-risk groups. For instance, 5-aminoimidazole-4-carboxamide-1-β-riboside (AICAr) and BMS.754,807 had higher drug sensitivity in a low-risk group than in a high-risk group, while Bleomycin had higher drug sensitivity in the high-risk group (Fig. 7A). We analyzed the enrichment of high- and low-risk samples in various clusters in the TCGA dataset using the fisher test, and the results of the enrichment are displayed in (Fig. 7B). High-risk samples were significantly enriched in cluster 1, while low-risk samples were significantly enriched in cluster 2 and cluster 4.

### External validation of model genes

**Fig. 8:**
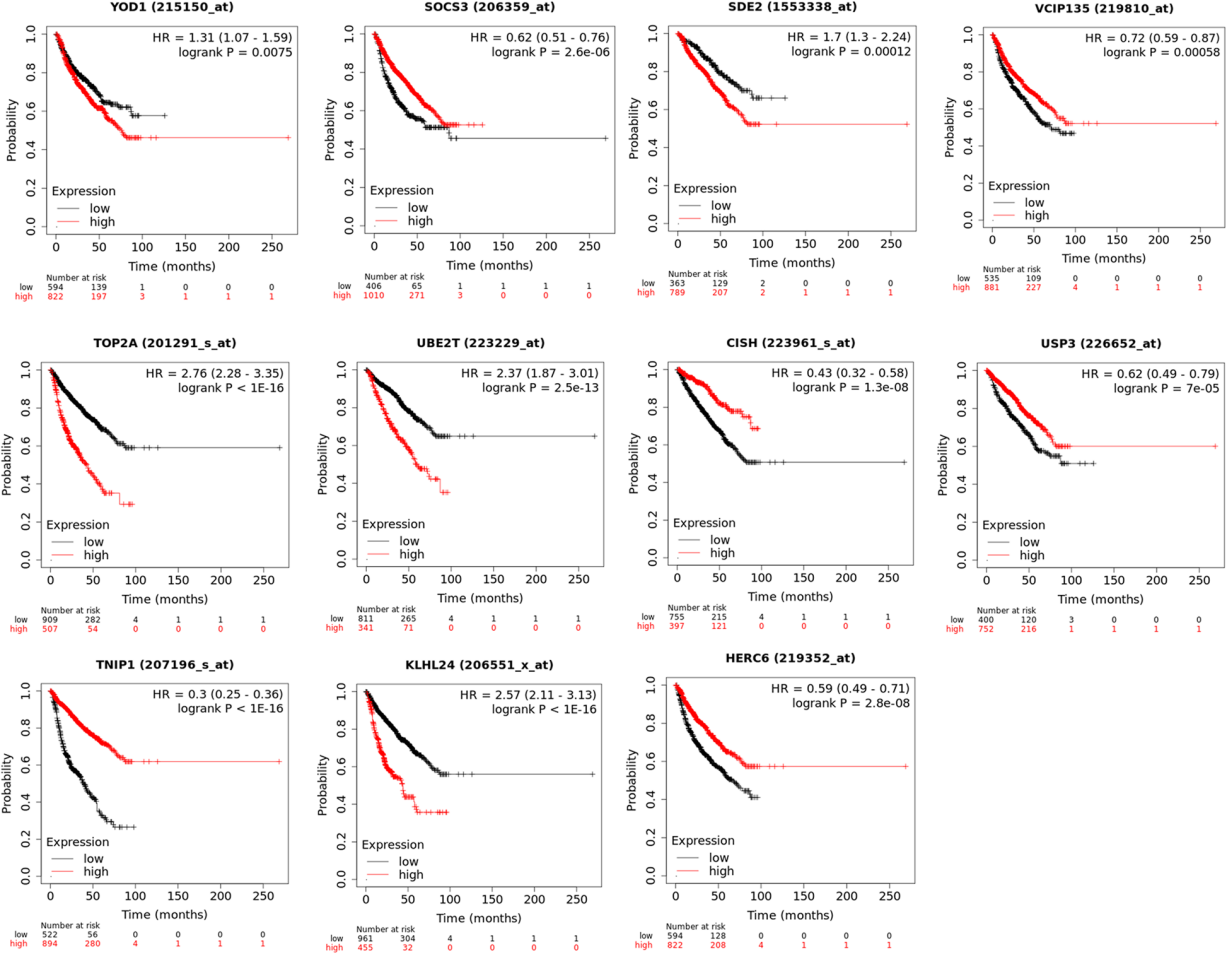
Correlation between the expression of model genes and survival of MM patients

The validation of these 11 model genes on the Kaplan-Meier Plotter database indicated that the expression of the model genes was significantly correlated with the survival of MM patients (*P* < 0.05, Fig. 8).

## Discussion

Protein modification plays a crucial role in the regulating of cellular processes, in which protein ubiquitination can alter tumor metabolism and immune regulation [24]. Ubiquitination is related to the stability of oncoproteins in MM [25]. Nowadays, the proteasome inhibitor has been widely used in treatment of MM [11, 26]. However, drug resistance is one of the major challenges we face. And the correlation between ubiquitination-related genes and MM has not been thoroughly examined.

According to the DEGs analysis, a total of 47 ubiquitination-related genes were highly expressed in the MM group, while 40 genes were highly expressed in the control group, indicating a connection between ubiquitination-related genes and MM. Four clusters exhibited significant differences according to the consistent cluster analysis. At the same time, the survival information was significantly among different clusters. The prognosis of cluster 1 was poor, and the enrichment score was significantly lower than that of cluster 2 and cluster 4. It is suggested that the genes related to to ubiquitination are associated with the prognosis of MM patients. Patients with poor survival may experience early death. Developing targeted therapies that act on genes associated with aggressive disease or drug resistance can be applied to improve outcomes for MM patients with high-risk [27]. The tumor purity in clusters 1 and 3 was higher than that in clusters 2 and 4 consistent with the trend of GSAV score.

In this study, the analysis of gene expression levels of using TCGA and GEO datasets revealed that the that model genes UBE2T, TOP2A, VCPIP1, SDE2, YOD1, and SOCS3 were highly expressed in the low-risk group, whereas KLHL24, HERC6, USP3, TNIP1, and CISH were highly expressed in the high-risk group. Regarding genes highly expressed in high-risk groups, KLHL24 is the main substrate adaptor protein of Cullin3-RING ligase (CRL3) which is one of the most common E3 ubiquitin ligases. CRL3 can affect the stability of functional proteins by mediating substrate ubiquitination modifications. Dysregulation of CRL3 can lead to the development of multiple diseases. KLHL24 inactivation is associated with hypertrophic cardiomyopathy [28, 29]. KLHL24 inhibits the activation of fibroblasts, leading to skin fibrosis, and hinders skin wound healing [30]. People who carry a mutation in the KLHL24 gene are at risk of developing epidermolysis bullosa [31]. HERC6 is also an E3 ubiquitin ligase, primarily expressed in the testicles and fetal brain, with rare expression in the heart and skeletal muscle. HERC6 participates in a variety of cellular activities, including cell proliferation, cell migration, and neurodevelopment. HERC6 can be cancer-promoting factors and tumor suppressor genes, depending on the type of cancer [32]. At the same time, the up-regulation of HERC6 is present in systemic lupus erythematosus (SLE) and promotes inflammation [33]. Ubiquitin-specific protease 3 (USP3) is highly expressed in multiple cancers. It plays an important role in tumor proliferation and invasion. The overexpression of USP3 leads to an unfavorable prognosis in breast cancer patients and stomach cancer metastasis [34]. Furthermore, the activation of USP3 induces neuroblastoma [35]. The tumor necrosis factor α-induced protein 3-interacting protein 1 (TNIP1) plays a part in mitophagy [36]. It has an impact on the development of autoimmune diseases [37], such as lupus nephritis [38]. The overexpression of cytokine-inducible SH2-containing protein (CISH) promotes inflammation in the elderly [39]. The knockdown of CISH may regulate the metabolic activity of NK cells to exert an anti-tumor effect [40, 41]. In addition, a previous study has shown that the inhibiting of CISH could improve the outcomes of immune checkpoint blockade therapy [42]. The existing study has confirmed that HERC4 is involved in inhibiting the proliferation of myeloma cells [43]. More important, model genes were verified to be significantly correlated with the survival of MM patients through external validation. The role of KLHL24, HERC6, USP3, TNIP1, and CISH genes in MM has not been reported in the literature, so it is worth further investigation in vitro and in vivo.

Effector lymphocyte dysfunction and malignant plasma cells are associated with MM which is accompanied by immunosuppression [44]. Immune escape and loss of antigenicity appear in MM. Tumor cells in patients with MM exhibit increased expression of the immune checkpoint receptor programmed death receptor ligand that promotes immune escape [45]. The proportions of B cells memory, plasma cells, T cells CD4 naïve, and T cells CD8 showed significant differences between the clusters in this study. In addition, the differences in ICGs and HLA genes between the clusters were statistically significant, except for BTN2A2, HLA-DQA1, and HLA-DRB5. After the intersection, a total of 72 ubiquitination representative genes were screened and divided into high- and low-risk groups for survival analysis.

In the present study, AICAr exhibited higher drug sensitivity in a low-risk group. It has been reported the effects in accelerating apoptosis [46] and inhibits the growth of MM cells [47]. Insulin-like growth factor 1 receptor/insulin receptor family kinases inhibitor BMS.754,807 also showed higher drug sensitivity in the low-risk group in this study. The evidence showed that it is effective in inhibiting MM cells [48]. Bleomycin demonstrated higher drug sensitivity in the high-risk group in the present study. It could be used in drug-resistant MM patients, which plays a role in the treatment of drug-resistant MM [49]. In Fig. 2E, cluser4 is clearly distinguished by PCA, but cluster1 \ 2 \ 3 has significant overlap. Exploring additional biological features related to gene clusters of these different clustering patterns, in order to identify patient sub clusters and develop therapies in clinical, will be a highly promising work in the future. Although this study identified ubiquitination genes associated with multiple myeloma, But differences in the transcriptome of hematopoietic system tumors may result in variations in drug efficacy among different samples and developmental stages [50]. Additionally, this study lacks analysis based on ubiquitination-related genes, risk scores, and drug sensitivity across samples at different developmental stages, which is a potential research point.

The risk model had exhibited satisfactory predictive power. It was elucidated that there was poor survival in the high-risk group. Furthermore, genes related to ubiquitination were initially explored in connection with MM in our study. Ubiquitination has a significant impact on celluar protein interactions and is linked to cancer progression. Due to the wide range of viral and non viral etiologies in MM [51, 52], clustering based on ubiquitination can better distinguish the differences in etiology of multiple myeloma, identify different interaction patterns between these clusters, and provide personalized treatment for patients based on these etiologies.

Whereas, there were some limitations in the present study. First, we did not find normal bone marrow samples in the same dataset but screened control samples in the leukemia dataset, and there is a lack of ethnic details in clinical data, which may have led to a decrease in reliability. Furthermore, we lacked survival information other than survival time and death time for LASSO regression analysis to enhance the effectiveness of the risk model. Due to the fact that only a very small number of enriched immune cells show insignificant differences in immune scores across different clusters, validation of all immune cells is necessary. However, due to limitations in research conditions, we are unable to conduct cell experiments for validation. This is a limitation of the study and we hope to verify it in future research.

## Conclusion

The risk model is constructed to predict the prognosis of MM patients. Overexpression of ubiquitination-related genes such as KLHL24, HERC6, USP3, TNIP1, and CISH may indicated a poor prognosis and lower survival rate in MM patients. These genes have the potential as therapeutic targets for MM. The mechanisms of these genes in drug sensitivity and MM pathogenesis deserve further study.

### Supplementary Information

Supplementary Material 1.

Supplementary Material 2.

Supplementary Material 3.

Supplementary Material 4.

Supplementary Material 5.

## Data Availability

The data in this study are available with permission from the corresponding author.
