# Supplementary material for: The prognostic significance of ubiquitination-related genes in multiple myeloma by bioinformatics analysis
Source: BMC Med Genomics. 2024 Jun 19;17:164. doi: 10.1186/s12920-024-01937-0 (PMC11186196; doi:10.1186/s12920-024-01937-0)
Supplement: Supplementary file 5 — Supplementary Material 5. [file 12920_2024_1937_MOESM5_ESM.docx]

**Table 24.** The correlation between risk score and survival time by single factor analysis

|  | coef | exp(coef) | se(coef) | z | Pr(>\|z\|) |
| --- | --- | --- | --- | --- | --- |
| riskscore | 186208.8 | Inf | 37907.87 | 4.91214 | 9.008759e-07 |
